# Gut microbiome and metabolomics in systemic sclerosis: feature, link and mechanisms

**DOI:** 10.3389/fimmu.2024.1475528

**Published:** 2024-11-04

**Authors:** Qicen Yao, Wenfeng Tan, Feihu Bai

**Affiliations:** ^1^ Department of Rheumatology and Immunology, The Second Affiliated Hospital of Hainan Medical University, Haikou, China; ^2^ Nanjing Medical University, Nanjing, China; ^3^ Department of Rheumatology, The First Affiliated Hospital of Nanjing Medical University, Nanjing, China; ^4^ Department of Gastroenterology, The Second Affiliated Hospital of Hainan Medical University, Haikou, China

**Keywords:** systemic sclerosis, gut microbiota dysbiosis, metabolites, correlation analysis, pathogenesis

## Abstract

Systemic sclerosis (SSc) is a rare and highly heterogeneous chronic autoimmune disease characterized by multi-organ and tissue fibrosis, often accompanied by a poor prognosis and high mortality rates. The primary pathogenic mechanisms of SSc are considered to involve tissue fibrosis, autoimmune dysfunction, and microvascular abnormalities. Recent studies have shed light on the gut microbiota (GM) and metabolites in SSc patients, revealing their association with gastrointestinal symptoms and disease phenotypes. However, further elucidation is needed on the specific mechanisms underlying the interactions between GM, metabolites, and the immune system and their roles in the pathogenesis of SSc. This review outlines the characteristics of GM and metabolites in SSc patients, exploring their interrelationships and analyzing their correlations with the clinical phenotypes of SSc. The findings indicate that while the α-diversity of GM in SSc patients resembles that of healthy individuals, notable differences exist in the β-diversity and the abundance of specific bacterial genera, which are closely linked to gastrointestinal symptoms. Moreover, alterations in the levels of amino acids and lipid metabolites in SSc patients are prominently observed and significantly associated with clinical phenotypes. Furthermore, this review delves into the potential immunopathological mechanisms of GM and metabolites in SSc, emphasizing the critical role of interactions between GM, metabolites, and the immune system in comprehending the immunopathological processes of SSc. These insights may offer new scientific evidence for the development of future treatment strategies.

## 1 Introduction

Systemic sclerosis (SSc) is a rare and complex chronic autoimmune disease that causes fibrosis in multiple organs, particularly the skin and lungs (1). Among the skin lesions associated with SSc, the most common types are limited cutaneous SSc (lcSSc) and diffused cutaneous SSc (dcSSc). Despite these common typical presentations, SSc displays a broad range of clinical manifestations and diverse prognostic features, holding the highest mortality rate among all rheumatic diseases (2). A comprehensive study by EUSTAR (EULAR Scleroderma Trials and Research) revealed that pulmonary fibrosis (PF) is responsible for 35% of SSc-related deaths, while pulmonary arterial hypertension (PAH) and cardiac complications each account for 26% (3). Therefore, the extensive multi-organ and tissue fibrosis caused by SSc, along with its poor prognosis and high mortality rates, severely affect patients’ quality of life and overall survival. Additionally, the SCORE project’s multicenter study highlighted that gastrointestinal tract (GIT) complications are the third most common cause of death in SSc patients, following PAH and interstitial lung disease (ILD) (4). Notably, about 90% of SSc patients experience some degree of GIT fibrosis during the disease course (3). Thus, the widespread multi-organ and tissue fibrosis secondary to SSc, coupled with its poor prognosis and elevated mortality rates, significantly impact patients’ quality of life and overall survival.

The pathogenesis of SSc remains complex and multifactorial, involving tissue fibrosis, autoimmune dysfunction, and microvascular abnormalities (4). Key cell types such as immune cells, fibroblasts, and endothelial cells, alongside the inflammatory mediators, are central to the initiation and progression of SSc (5–7). Recent research has highlighted the role of the gut microbiota (GM) in maintaining host health through interactions with environmental factors, genetic predispositions, metabolites, and immune signals (8, 9). Disruptions in the GM and their metabolites have been implicated as potential triggers for the development or progression of SSc and associated multi-organ damage (10–14), closely intertwined with inflammatory responses, vascular impairment, and fibrotic processes (11). The evolution of multi-omics and high-throughput sequencing technologies has significantly enriched our comprehension of the actions of GM and metabolomics in the host (15, 16). However, investigations into the relationship between the two realms remain somewhat limited. Hence, further investigation into the interplay between GM, metabolites, and the immune system holds significant importance in elucidating the immunopathological mechanisms underlying SSc.

This review aims to synthesize the most recent research advancements, elucidate the methodologies and features of detecting GM and metabolites in SSc, and better understand the interconnection between GM and metabolites, along with their possible mechanisms of influence in SSc. By conducting this thorough analysis, our goal is to offer a fresh insight into the pathophysiology of SSc and establish a sound scientific foundation for forthcoming therapeutic approaches.

## 2 Alterations in the intestinal microenvironment of SSc patients and their clinical relevance

The intestinal microbiota has emerged as a focal point of research in SSc, with numerous studies highlighting the presence of microbial imbalance in SSc patients (**Table 1**). These imbalance have shown significant correlations with disease progression, subtypes, prognosis, and treatment outcomes in SSc. Additionally, abnormalities in the microbiota composition have been observed at fibrotic sites, such as the skin and intestines, in SSc (17). However, the presence of a lung microbiome remains controversial. To date, only a few studies have explored the relationship between the gut microbiome and SSs-ILD. Interestingly, the bronchoalveolar lavage fluid from patients with rheumatoid arthritis-associated ILD showed significantly lower microbial diverse and abundant (18). These findings indicate a potential association between GM’s dysregulation and SSc’s pathogenesis.

**Table 1:** Alterations of the gut microbiome in SSc.

Year	Region	Subjects (n)	Sample type	Methods	Gut microbiota in patients with SSc	Reference
2016	Sweden	SSc (98)	Feces	GA-map™ Dysbiosis Test	Faecalibacterium prausnitzii and Clostridiaceae and Lactobacillus↑.	(29)
2016	United States	SSc (17) VS HC (17)	Cecum and sigmoid mucosal lavage samples	16S rRNA	Commensal bacteria: Faecalibacterium and Clostridium ↓. Pathobiont bacteria: Fusobacterium and γ-Proteobacteria↑. Commensal genera: Bifidobacterium and Lactobacillus↑.	(22)
2017	Italy	SSc-GI+ (9) VS SSc-GI- (9) VS HC (9)	Feces	16S rRNA	SSc-GI+ vs HC: Lactobacillus, Eubacterium and Acinetobacter↑. Roseburia, Clostridium, and Ruminococcus↓. SSc-GI- vs HC: Streptococcus salivarius↑.	(28)
2017	United States, Norway	UCLA-SSc (17), OUH-SSc (17) VS HC (17)	Feces	16S rRNA	UCLA VS HC Phylum: Firmicutes↑, Bacteroidetes↓. Commensal genera: Fusobacterium, Ruminococcus, γ-Proteobacteria, Erwinia and Lactobacillus ↑, Faecalibacterium and Bacteroides ↓. OUH VS HC Phylum: Bacteroidetes↓. Commensal genera: Lactobacillus ↑, Clostridium, and Bacteroides ↓. OUH VS UCLA Commensal genera: Faecalibacterium and Bacteroides ↑.	(21)
2018	Italy	SSc (59) VS HC (28)	Feces	16S rRNA	Parabacterioides, Firmicutes, Butyricimonas, and Desulfovibrio↑. Turicibacter and Lachnospiraceae ↓.	(20)
2020	Italy	SSc (63) VS HC (17)	Feces	16S rRNA	Lactobacillus and Streptococcus↑. Sutterella↓.	(19)
2020	USA	SSc (19)	Feces	16S rRNA	Genus: Clostridiales↑, Bacteroides↓.	(23)
2021	Canada	SSc (29) VS HC (20)	Feces	16S rRNA	phyla: Proteobacteria and Bacteroidetes ↑, Firmicutes↓. Genus: Bacteroides and Lachnospira↑, Enterococcus, and Lactococcus↓.	(26)
2021	United States	SSc (90) and IgG4-RD (58) VS HC (165)	Feces	Metagenomic sequencing	SSc and IgG4-RD VS HC pathogenic Clostridium and oral Streptococcus↑, Alistipes and Bacteroides↓.	(31)
2022	United States, Sweden	UCLA-SSc (71), LU-SSc (106) VS HC (85)	Feces	16S rRNA	UCLA VS HC pathobionts Streptococcus, Enterococcus↑, LU VS HC pathobiont genera: Desulfovibrio ↑ commensal genera: Faecalibacterium ↓.	(30)
2023	Singapore	SSc (23) VS HC (19)	Feces	Metagenomic sequencing	Phyla: Firmicutes, Actinobacteria↑, Bacteroidetes↓. Genus: Lactobacillus, Bifidobacterium and Coprococcus↑	(10)
2023	United States	SSc (66)	Feces	16S rRNA	Non-low FODMAP diet group: pathobiont Enterococcus↑. Severe GI symptoms group: Lactobacillus and Firmicutes↑.	(25)
2024	Italy	SSc (25)	Feces	16S rRNA	SSc Phyla(top 5): Proteobacteria, Firmicutes, Actinobacteria, Bacteroidetes, and Verrucomicrobia↑.	(24)
					ACA+ VS anti-Scl70+: Phylum: Lentisphaerae↑. Genus: NA-Acidaminococcaceae↑. Classes: Lentisphaeria and Opitutae↑.	

↑ increased, ↓ decreased. HC, healthy controls; 16S rRNA, 16S ribosomal RNA sequencing; GI+, gastrointestinal involvement; UCLA, the University of California, Los Angeles; OUH, Oslo University Hospital; IgG4-RD, immunoglobulin G4-related disease; LU, Lund University; FODMAP, a low versus non-low fermentable oligosaccharides, disaccharides, monosaccharides, and polyols; ACA, anticentromere antibody.

### 2.1 Alterations of the GM in SSc

Recent research indicates that the α diversity in SSc patients mirrors that of healthy controls (HC) (19–22). Moreover, a year-long longitudinal study revealed no notable alterations in α and β diversity or the relative abundance of GM throughout the disease progression in SSc patients (23). However, variations exist in the abundance of different taxa within the GM. At the phylum level, Firmicutes (10, 20, 21, 24, 25), Proteobacteria (21, 22, 24, 26), and Actinobacteria (10, 24) exhibit increased abundance, while Bacteroidetes display a fluctuating trend of abundance (10, 21, 24, 26). Additionally, a higher relative abundance of Fusobacteria has been noted (24). These observations underscore distinct differences in the GM composition between SSc patients and the healthy population (27). At the genus level, several genera, including Lactobacillus (10, 19, 21, 22, 25, 28, 29), Streptococcus (19, 28, 30, 31), Fusobacterium (21, 22), Enterococcus (25, 30), Desulfovibrio (20, 30), Faecalibacterium (29), and Bacteroides (26) show elevated relative abundances in SSc patients. Conversely, commensal genera associated with anti-inflammatory effects, such as Faecalibacterium (21, 22, 30) and Bacteroides (21, 23, 31), exhibit reduced relative abundance. Clostridium, known for inducing the expansion of regulatory T cells (32), also shows decreased relative abundance in SSc patients (21, 22, 28). On the other hand, pathogenic Clostridium (31) and Bacteroides (26) exhibit increased relative abundance, while Roseburia (28) shows a decline. These shifts in the abundance of various microbial genera, including Faecalibacterium (21, 22, 30) and Clostridium (21, 22, 28), suggest a potential association between GM abnormalities and immunoinflammation in SSc.

Furthermore, intriguing trends in the relative abundance of Bifidobacterium and Lactobacillus (34) in SSc contrast with patterns typically observed in inflammatory bowel disease. For instance, Lactobacillus (12, 20, 22, 23, 26, 29, 30) and Bifidobacterium (12, 23) exhibit elevated relative abundances. Specifically, two independent studies reported a significant increase in Lactobacillus counts among SSc patients (22). Additionally, a separate cohort study observed a higher abundance of commensal bacteria, specifically Bifidobacteria, in the University of California Los Angeles cohort than in the Oslo University Hospital cohort (30). The study suggested that genetic factors, dietary patterns, or the presence of SSc-associated ILD may play a role in this diversity among cohorts. Moreover, Natalello et al. highlighted elevated levels of Lactobacillus and Streptococcus in SSc patients compared to healthy controls (HCs), alongside reduced expression of Sartellabacteria, which is negatively associated with inflammation (19). The genus Ruminalococcus displayed variable abundance levels across studies (21, 28). These findings suggest a potential correlation between GM abnormalities and immunoinflammation in SSc.

The GM has been implicated in the pathogenesis and clinical manifestations of various rheumatic diseases, contributing to fibrotic processes in specific internal organs (33). Previous evidence has indicated a decrease in GM diversity in SSc patients, notably in those who are overweight and have a disease duration exceeding three years (19). Research conducted by Andreasson et al. proposed a potential association between GM richness and the progression of SSc, ILD, small intestinal bacterial overgrowth (SIBO), and the utilization of immunosuppressants (30). Additionally, GM is believed to influence immune cell activity in SSc, with variations in bacterial flora abundance observed between SSc patients positive for anticentromere antibody (ACA) and those positive for anti-Scl-70 (anti-DNA topoisomerase I) (24). A study exploring the connection between SSc and immunoglobulin G4-related disease (IgG4-RD), which predisposes individuals to fibrotic disorders, revealed heightened levels of pathogenic Clostridium difficile and typical oral streptococci in comparison to HC (31). The study also highlighted an increase in E. lenta cgr+ strains capable of activating Th17, while homocysteine (Hcy)-producing Clostridium difficile displayed a preferential colonization in SSc. Concurrently, reductions in Alistipes, Bacteroides, and species producing butyrate were noted in both diseases (31). These alterations imply a decrease in beneficial commensal species and an increase in potentially harmful and proinflammatory strains that may influence inflammatory and fibrotic processes by modulating Th17 cell responses. While the literature has progressively reported on the association between GM and SSc subtypes, there are conflicting studies suggesting that ecological dysbiosis is not linked to age, disease duration, disease subtype, or the extent of dermal fibrosis (29). Therefore, the predominant GM profile in SSc remains ambiguous, and its relationship with disease subtypes and fibrosis continues to be discussed and investigated.

### 2.2 Association of GM dysbiosis with gastrointestinal symptoms in SSc

Despite GIT involvement being prevalent in up to 90% of SSc patients, most exhibit unremarkable symptoms with a diverse clinical presentation. The UCLA Scleroderma Clinical Trials Consortium’s Gastrointestinal Tract Scoring Tool 2.0 (UCLA-GIT 2.0) is a widely utilized tool for evaluating GIT involvement in SSc (34). Dysregulation of GM and SSc-related GIT symptoms can manifest at any stage of the disease (30). Research revealed that SSc patients with GIT symptoms (SSc/GIT+) displayed a significantly lower α diversity index compared to both HCs and SSc patients without GIT symptoms (SSc/GIT-). Moreover, SSc/GIT+ patients exhibited reduced fecal microbiota abundance yet higher homogeneity (28). However, β-diversity analyses indicated greater variability in fecal microbiota among SSc/GIT+ patients (28). Specifically, a decrease in Bacteroides abundance was correlated with the duration of GIT symptoms (23). Esophageal dysfunction, commonly seen in SSc-GIT, may present as reflux disease with or without esophagitis (29). In addition, about 50% of SSc patients experience lower GIT involvement, which correlates with increased morbidity and mortality, showcasing clinical signs such as malabsorption, constipation, diarrhea, recurrent pseudo-obstruction, and fecal incontinence (1). Remarkably, SSc patients with concurrent SIBO exhibit higher bacterial diversity and abundance. However, there was no significant symptom discrepancy between SIBO-positive and SIBO-negative patients that could be independently linked to microbial composition (26, 30).

Recent studies have validated the association between gastrointestinal symptoms and GM imbalances. Specifically, an escalation in the abundance of pathogenic genera such as Klebsiella and Enterococcus was noted among patients with more severe symptoms (25). In the aforementioned independent cohort study involving SSc patients, it was observed that Clostridium was prevalent in those with lower gastrointestinal symptom severity, while Lactobacillus levels were higher in individuals experiencing none-to-mild constipation. Additionally, Prevotella showed heightened presence in patients with moderate-to-severe gastrointestinal symptom severity (21). Lactobacillus, a widely recognized probiotic strain crucial for maintaining GM equilibrium, has consistently shown increased levels in SSc patients across various studies (10, 19, 21, 22, 25, 28, 29), suggesting its potential role in alleviating gastrointestinal symptoms in SSc. Conversely, a separate investigation highlighted a reduction in *Bacteroides fragilis* abundance and an elevation in Fusobacterium levels among SSc patients with moderate to severe gastrointestinal symptoms (22). These findings underscore a probable correlation between specific microbial flora and the intensity of gastrointestinal symptoms in patients, offering prospective microbial targets for future therapeutic interventions.

## 3 Alterations of the metabolism in SSc

Recent literature reports significant changes in metabolite levels in the blood, fecal, and urine samples of SSc patients, with these alterations showing correlations with the SSc phenotype (11, 13). Nevertheless, our current knowledge regarding the specific functions of these metabolites in the pathophysiological pathways of SSc remains restricted. Therefore, a detailed examination of the advancements in metabolomics research related to SSc is crucial for a comprehensive comprehension of the pathophysiological mechanisms involved. Moreover, metabolomics is anticipated to offer valuable data to support SSc diagnosis, disease classification, personalized therapeutic approaches, as well as the identification and validation of biomarkers.

### 3.1 Alterations in metabolites and their metabolic pathways in SSc

Several studies have examined changes in the plasma, serum, and urine concentrations of amino acids and their derivatives or metabolites in SSc patients (**Table 2**). The findings reveal elevated levels of amino acids such as glutamine, proline, glutamate, alanine, arginine, and Hcy, alongside metabolites linked to amino acid derivatives (betaine, 1-methylhistidine, 3-methylhistidine, dimethylarginine, phenylacetylglutamine, methylnicotinamide, kynurenine, malondialdehyde) in the plasma/serum of SSc patients (35–43). Conversely, some studies have reported reduced levels of alanine, aspartate, glutamate, and L-tyrosine (42–44). Furthermore, various studies have noted decreased levels of tryptophan (Trp) in SSc patients (39, 41–43, 45), a finding corroborated in hypochlorite- and bleomycin-induced mouse models of SSc (45). Notably, kynurenine, a classic Trp metabolite, was found at heightened concentrations in the plasma and serum of SSc patients (39, 42). While some studies indicated higher plasma Hcy levels in SSc patients compared to HCs (36, 37), others reported no significant difference in Hcy levels between these groups (46, 47). Moreover, elevated levels of α-N-phenylacetyl-L-glutamine were detected in SSc patients, contrasting with decreased levels of proline betaine (48). Collectively, these findings suggest that amino acid dysregulation may play a crucial role in the metabolic pathways associated with SSc.

**Table 2:** Alterations of metabolites in SSc.

Year	Region	Subjects (n)	Sample type	Methods	Altered metabolites in SSc vs HC	References
2000	Sweden	SSc (27) VS HC (27)	Plasma,	GC-MS	Nitrate↑.	(115)
2003	Italy	SSc (71)VS HC (30)	Plasma	HPLC-FLD	Homocysteine↑.	(37)
2006	South Africa	SSc (15) VS HC (13)	Plasma	GC-MS	Malondialdehyde↑.	(35)
2007	Italy	SSc (60) VS HC (30)	Plasma	HPLC-FLD	Homocysteine↑.	(36)
2009	United States	SSc (10)VS HC (14)	Serum	HPLC-QTRAP-MS	Arachidonoyl-lysophosphatidic acid, sphingosine 1-phosphate↑.	(57)
2016	Sweden	SSc (19) VS HC (18)	Serum	GC-MS	arginine↑. 2-oxoglutaric acid↓.	(40)
2016	Italy	SSc (40) VS HC (40)	Plasma	HPLC-MS	25-hydroxivitamin D3 ↓.	(116)
2018	Italy	SSc (37) VS HC (20)	Serum	H-NMRS and GC-MS	Glutamine, 3-OH-butyrate↑. Citrate, aspartate, alanine, choline, glutamate, glutarate, glycerate, and threonate↓.	(38)
2018	Italy	SSc (59) VS HC (28)	Plasma	UHPLC-Q-TOF-MS	DL-2-aminooctanoic acid, Diacylglycerol 38:5, 1-(9Zpentadecenoyl)-glycero-3-phosphate, phosphatidylcholine 36:4, 2,4-dinitrobenzenesulfonic acid, alpha-N-phenylacetyl-l-gl↑.	(20)
2019	United Kingdom	SSc (97) VS HC (10)	Serum	HPLC-FLD	Kynurenine↑. Tryptophan↓.	(39)
2019	Italy	SSc (59) VS HC (28)	Plasma, Urine	HPLC-ESI-QTOF-MS	Plasma: Alpha-N-phenyl acetyl-L-glutamine, Butyrylcarnitine, Valerylcarnitine, 2-4-dinitrobenzenesulfonic acid, Oleic acid, 1arachidonoylglycerol monoacylglycerol, Monoacylglycerol↑. Urine: D-Sorbitol, N-cyclohexylformamide, Ser-Pro-Pro, Dihydroxy1H-indole glucuronide, 2-(2-Phenylacetoxy)propinylglycine, Alpha-Nphenylacetyl-L glutamine, Pyroglutamic acid↑. N-Methylnicotinamide, Proline betaine, Creatinine, Vinylacetylglycine, N1-methyl-4pyridine-3-carboxamide, N1-methyl-2pyridine-5-carboxamide, Hydroxyprolyl-valine, L-beta-aspartyl-L-Leucine, Hypaphorine, 2-octanoyl-carnitine, Decatrienoylcarnitine, 2-Nonenoylcarnitine, 2,6-Dimethylheptanoyl carnitine, 9-Decenoylcarnitine, Hydroxydodecenoylcarnitine, Undecenoyl carnitine↓.	(48)
2020	Switzerl	SSc (36) VS HC (12)	Serum	UHPLC-Q-TOF-MS	1-methyladenosine↑. L-tryptophan, L-tyrosine↓.	(43)
2020	Italy	SSc (20) VS HC (7)	Plasma	UHPLC-Orbitrap-MS	Lauric acid, myristic acid, arachidic acid, carnitine, isovalerylcarnitine↑. Octanoyl-carnitine, palmitoyl-carnitine↓.	(54)
2020	Poland	SSc (42) VS HC (27)	Plasma	LC/MS	Glutamine, proline, 1-methylhistidine, betaine, methylnicotinamide, asymmetric dimethylarginine↑. Tryptophan↓.	(41)
2021	Austria	SSc (58) VS HC (48)	Plasma	HPLC-IM-Q-TOF-MS	Phosphatidylcholine 34:1, 34:2, 34:3; sphingomyelin 33:1, 35:1, 35:2↑.	(56)
2022	Austria	SSc (52) VS HC (48)	Plasma	HPLC-IM-Q-TOF-MS	Kynurenine, dimethylarginine, citrulline, ornithine, phenylacetylglutamine, 1-methylhistidine, 3-methyl↑. Tryptophan, OH-tryptophan, alanine, lysophosphatidylcholine 22:4a, 22:4b, 20:2; sphingomyelin 34:1, 40:3↓.	(42)
2023	China	SSc (30) VS HC (30)	Serum	HILIC UHPLC-Q-TOF MS	Vitamin E, Alpha-N-phenylacetyl-L-glutamine, L-glutamine, L-isoleucine, phenol, 2-oxoadipic acid, 1-palmitoyl-2-hydroxy-sn, glycero-3-phosphoethanolamine, Chenodeoxycholate, Indoxyl sulfate, D-quinovose↑. Confertifoline, Azelaic acid, Vanillin, 3b-hydroxy-5-cholenoic acid, 1-stearoyl-sn-glycerol, Magnolol, Trans-dehydroandrosterone, 4-Nonylphenol, Norethindrone acetate, cis-9,10-epoxystearic acid, 16-Hydroxypalmitic acid, 2-Ethyl-2-hydroxybutyric acid, Stearic acid, Hexadecanedioic acid, Embelin, 3-Hydroxycapric acid, Androsterone sulfate, Benzenebutanoic acid, Pregnenolone sulfate, Arachidonic acid, Dodecanoic acid, Palmitic acid, myristic acid, Cholesterol 3-sulfate, Caprylic acid, Cis- (6,9,12)-linolenic acid, Alpha-ketocaproic acid↓.	(44)
2023	Poland	SSc (63) VS HC (47)	Plasma	HPLC-MS	Trimethylamine N-oxide (TMAO) ↑.	(60)
2023	Poland	SSc (50) VS HC (30)	Serum	ELISA	Markers of Intestinal Permeability: lipopolysaccharides↑.	(59)
2023	China	SSc baseline (127)VS HC(30)	Serum	HPLC-Q-TOF-MS	Hydroxyisocaproic acid, citric acid, isobutyric acid, phloretin 2’-O-glucuronide, and thromboxane A2↑. 5-methoxytryptophol, 12 (13)Ep-9-KODE, alpha-tocotrienol, chlorogenic acid, and cholic acid glucuronide↓.	(45)
		SSc treatment (57) VS HC (30)			Amidosulfonic acid, L-proline, L-glutamic acid and betaine↑. Uracil, phthalic acid, guanidinosuccinic acid, and isovalerylglycine↓.	

↑ increased, ↓ decreased. GC-MS, Gas chromatography-mass spectrometry; HPLC-FLD, High-performance liquid chromatography with fluorescence detection; HPLC-QTRAP-MS, High-performance liquid chromatography quadrupole-linear ion trap hybrid mass spectrometry; HPLC-MS, High-performance liquid chromatography-mass spectrometry; H-NMRS and GC-MS, H-Nuclear Magnetic Resonance Spectroscopy and Gas Chromatography-Mass Spectrometry; UHPLC-Q-TOF-MS, Ultra-high-performance liquid chromatography quadrupole time-of-flight mass spectrometry; HPLC-ESI-QTOF-MS, High-performance liquid chromatography coupled to electrospray ionization and quadrupole time-of-flight mass spectrometry; UHPLC-Orbitrap-MS, Ultra-high-performance liquid chromatography coupled with ion trap mass spectrometry; LC/MS, liquid chromatography/mass spectrometry; HPLC-IM-Q-TOF-MS, High-performance liquid chromatography coupled to ion mobility quadrupole time-of-flight mass spectrometry; HILIC-UHPLC-Q-TOF MS, Hydrophilic Interaction Liquid Chromatography-UHPLC-Q-TOF MS; ELISA, Enzyme-Linked Immunosorbent Assay; HPLC-Q-TOF-MS, High-performance liquid chromatography quadrupole time-of-flight mass spectrometry.

Phospholipids are essential components for constructing cell and organelle membranes, and they play various cellular roles, including regulating cell shape, migration, and intercellular interactions (49, 50). Phospholipid signaling pathways are highly complex, and even minor changes in phospholipid levels can significantly impact cell survival (49). Studies have demonstrated the involvement of phospholipids in a range of diseases, including neurodegenerative and metabolic disorders, cancer, as well as inflammatory and autoimmune conditions (49, 51–53). Notably, variations in serum lipid content have been observed in SSc patients, with investigations primarily focusing on plasma and serum samples. Specifically concerning carnitine and its derivatives, studies have identified heightened concentrations of short-chain carnitines (e.g., carnitine, butyrylcarnitine, and acetylcarnitine) (48, 54), while long-chain fatty acid (FA)-related acylcarnitines (e.g., octanoyl-carnitine, palmitoyl-carnitine) exhibit a propensity for decrease in SSc patients compared to HCs (54). Carnitine plays a critical role in transporting FA into mitochondria for oxidation, which is vital for cellular energy metabolism (55). Furthermore, acylcarnitine levels in urine samples from SSc patients are lower than those in HCs (48). In the realm of FA and metabolites in SSc, diversity exists with no discernible trends. Some studies indicate elevated plasma levels of specific FAs, such as saturated FAs (e.g., lauric, myristic, arachidic acids) and unsaturated Fas (54), while others report a decrease in myristic acid levels (44). Additionally, *glycerolipids* (e.g., diacylglycerol and monoacylglycerol) exhibit an increasing tendency (20, 48). Glycerol phospholipids (e.g., arachidonoyl-lysophosphatidic acid, phosphatidylcholine) have demonstrated elevated levels (20, 56, 57), contrasting with the down-regulation of lysophosphatidylcholine and phosphatidylethanolamine (42, 56). Moreover, sphingomyelin levels were noted to be elevated among SSc patients in the context of sphingolipids (56). In urine analyses, saccharides (e.g., D-sorbitol) were found to increase, while levels of carnitine and its derivatives showed a decrease (48). Collectively, these findings underscore lipid metabolism disorders as a vital metabolic pathway in the progression of SSc.

Regarding other metabolites and metabolic pathways in serum, reductions were observed in levels of citrate within the tricarboxylic acid cycle metabolites, glycerate in the glycolytic pathway (38), 2-ketoglutarate within the tricarboxylic acid cycle (40). Lipopolysaccharides (LPS), metabolic byproducts of bacteria, are closely linked with intestinal inflammation (58). The early stages of SSc are typically characterized by inflammatory changes, with the intestinal inflammatory state potentially impacting mucosal permeability. Furthermore, a cross-sectional investigation identified a significant elevation in LPS levels in the serum of early-stage SSc patients (59), implicating LPS in the early intestinal inflammatory state of SSc. Additionally, various other metabolites exhibited alterations, including heightened levels of 3-OH-butyrate, pyrimidine, and trimethylamine N-oxide (TMAO) (38, 45, 60) and reduced levels of choline and glutarate (44). Aida et al. discovered that estradiol upregulated fibronectin expression in human dermal fibroblasts and induced dermal fibrosis *in vitro* (61).

### 3.2 Metabolic alterations in the clinical subtypes of SSc

Significant variations in metabolites exist among the clinical subtypes of SSc (**Table 3**) (11, 41–44). In terms of amino acid metabolism, patients with dcSSc exhibited notably higher levels of valine, glutamate, and lysine, alongside decreased levels of glutamine compared to lcSSc and HCs (38). Furthermore, derivatives linked to amino acid metabolisms, such as betaine (44), Kyn (42), 1-methylhistidine, and phenylacetyl glutamine (42), were elevated, while Trp levels decreased (39, 42). Notably, the Trp/Kyn ratio was highest in dcSSc patients positive for antiribonucleoprotein antibody ARA (39). In terms of lipid metabolism, individuals in the dcSSc group demonstrated elevated levels of lecithin (32:0) and reduced levels of phosphatidylethanolamine (38:5, 38:6), sphingomyelin (32:2, 40:4, 30:1) (56) and lysophosphatidylcholine (22:4) (42). Organic acid metabolism analysis revealed significantly increased levels of acetic acid, fructose, glycerol, glycerophosphate, and glutaric acid in dcSSc patients (38), while sorbitol, glucose, and lactate levels decreased (38). Moreover, a noteworthy finding was the presence of the NOS inhibitor L-asymmetric dimethylarginine (L-NAME) in patients with dcSSc or capillary dilatation, potentially indicating an association with vascular endothelial dysfunction and microangiopathy (41). Elevated Hcy levels may contribute to endothelial injury complexity in SSc, with plasma Hcy concentrations correlating with microvascular involvement and increasing with the progression of nailfold capillaroscopy patterns (36), subsequently leading to pulmonary vasculopathy secondary to ILD development (37, 46).

**Table 3:** Deregulation of metabolites in SSc subtypes.

Year	Region	Subjects (n)	Sample type	Methods	Altered metabolites in SSc VS HC	References
dcSSc and lcSSc						
2009	United States	dcSSc (7) VS lcSSc (3)	Serum	HPLC-QTRAP-MS	Sphingosine 1-phosphate↑.	(57)
2018	Italy	dcSSc (14) VS lcSSc (23)	Serum	H-NMRS and GC-MS	Valine, acetate, fructose, glutamate, glycerol, lysine, glycerate, glutarate↑. Sorbitol, glucose, lactate, glutamine↓.	(38)
2019	United Kingdom	dcSSc (58) VS lcSSc (39)	Serum	HPLC-FLD	Tryptophan↓.	(39)
2019	Italy	dcSSc (10) VS lcSSc (43)	Urine	HPLC-ESI-QTOF-MS	L-arogenate, N (5-amino-2hydroxybenzoyl)glycine, Indospicine↑. 3-methylglutarylcarnitine, 5-hydroxyindoleacetic acid↓.	(48)
2020	Poland	dcSSc (21) VS lcSSc (21)	Plasma	LC/MS	Sarcosine, beta-alanine, methylnicotinamide, N(G)nitro-L-arginine methyl ester (L-NAME)↑.	(41)
2021	Austria	dcSSc (11) VS lcSSc (39)	Plasma	HPLC-IM-Q-TOF-MS	Phosphatidylcholine 32:0↑. Phosphatidylethanolamine 38:5, 38:6, sphingomyelin 32:2, 40:4, 30:1↓.	(56)
2022	Austria	dcSSc (11) VS lcSSc (39)	Plasma	HPLC-IM-Q-TOF-MS	Kynurenine, citrulline, ornithine, Phenylacetylglutamine↑. Tryptophan, lysophosphatidylcholine 22:4↓.	(42)
2023	China	dcSSc (12) VS lcSSc (18)	Serum	HILIC UHPLC-Q-TOF MS	Trans-dehydroandrosterone, betaine, and SOPC↑. 1-palmitoyl-sn-glycero-3-phosphocholine↓.	(44)
SSc with ILD						
2014	Japan	SSc-ILD (65) VS SSc Non-ILD (86)	Plasma	HPLC-FLD	Homocysteine↑.	(46)
2019	Italy	SSc-ILD (18) VS SSc non-ILD (41)	Plasma	HPLC-ESI-QTOF-MS	N-(1-deoxy-1-fructosyl)-Valine, N-(1-deoxy-1-fructosyl)-leucine, N-(1-deoxy-1-fructosyl)-Isoleucine↑.	(48)
			Urine		Valyl valine, kynurenic acid, L-proline, proline-histidine, quinolinic acid, β-D-glucopyrapyranosil anthranilate↑.	
2020	Switzerland	Stable SSc-ILD (12) VS Progressive SSc-ILD (12)	Serum	UHPLC-Q-TOF-MS	L-Leucine, L-Isoleucine, Xanthosine↑. Adenosine monophosphate↓.	(43)
2023	China	SSc-ILD (19) VS SSc non-ILD (11)	Serum	HILIC UHPLC-Q-TOF MS	M-Glutamine↑. Ile-Ala, Androsterone sulfate↓.	(44)
2023	Poland	SSc-ILD (47) VS SSc non-ILD (16)	Plasma	HPLC-MS	Trimethylamine N-oxide (TMAO) ↑.	(60)
SSc with PAH						
2016	Australia	SSc-PAH (15) VS SSc Non-PAH (30)	Serum	HPLC-FLD	Asymmetric dimethylarginine, symmetric dimethylarginine↑. L-Arginine↓.	(65)
2017	Italy	SSc-PAH (8) VS SSc Non-PAH (10)	Plasma	H NMRS	Acetoacetate, Alanine, Lactate, VLDL, LDL↑. γ-Aminobutyrate, arginine, betaine, choline, creatinine, glucose, glutamate, glycine, histidine, phenylalanine, tyrosine↓.	(62)
2023	United States	PVDOMIC cohort: SSc-PAH (62) VS SSc non-PAH (19) VS HC (85)	Serum	LC-MS	kynurenine, N-acetylputrescine, kyn/trp and 1-methyladenosine↑.	(64)
		PVDOMIC cohort: SSc-PAH (32) VS HC (12)	Mixed and wedged venous		Kynurenine and kyn/trp↑.	
		JHSC cohort: SSc-PAH (81) VS HC (81)	Serum		Kynurenine/tryptophan (kyn/trp)↑.	
2023	United States	SSC-PAH (400) VS IPAH (1082)	Plasma	LC/MS	Fatty acid metabolism: Lignoceric acid fatty acyl esters of hydroxy fatty acid, nitrooleate, and Nervonic acid↑. Steroid hormones metabolism: 11-Testosterone and 17b estradiol↑. Arachidonic acid metabolism: Novel eicosanoid, prostaglandin F2a and leukotriene B4↑.	(63)
		SSc-PAH (44) VS SSc no-PAH (100)			SSc-PAH: Fatty acyl esters of hydroxy fatty acid, nervonic acid, 17b estradiol, prostaglandin F2a, And eicosanoid↑. SSc Non-PAH: Lignoceric acid and leukotriene B4↑.	
Autoantibodies are positive in SSc						
2019	United Kingdom	ARA + (47) VS ACA + (25) and Anti-Scl70 + (25)	Serum	HPLC-FLD	Kynurenine↑. Tryptophan↓.	(39)
2024	Italy	SSc (25)	Feces	GC-MS	Anti-Scl70 +: propionic acid↑. ACA +: hexanoic acid↑.	(24)
			Serum		ACA +:octanoic acids and octanoic acids↑.	
SSc with others						
2020	Poland	SSc calcinosis (6) VS SSc non-calcinosis (36)	Plasma	LC/MS	Glutamate, sarcosine, proline, tyrosine, 4-methylhistidine, ornithine↑.	(41)
		SSc JP (28) VS SSc non-JP (14)			Ornithine, 1-methylhistidine↑. Glutamine↓.	
		SSc-TE (24) VS SSc non-TE (18)			Glutamate, lysine, L-asymmetric dimethylarginine (NAME)↑.	
2023	Poland	SSc-ED (37) VS SSc non-ED (26)	Plasma	HPLC-MS	TMAO ↑.	(60)

↑ increased, ↓ decreased. dcSSc, diffused cutaneous SSc; lcSSc, limited cutaneous SSc; HPLC-QTRAP-MS, High-performance liquid chromatography quadrupole-linear ion trap hybrid mass spectrometry; H-NMRS and GC-MS, H-Nuclear Magnetic Resonance Spectroscopy and Gas Chromatography-Mass Spectrometry; HPLC-FLD, High-performance liquid chromatography with fluorescence detection; HPLC-ESI-QTOF-MS, High-performance liquid chromatography coupled to electrospray ionization and quadrupole time-of-flight mass spectrometry; LC/MS, liquid chromatography/mass spectrometry; LC/MS, liquid chromatography/mass spectrometry; HPLC-IM-Q-TOF-MS, High-performance liquid chromatography coupled to ion mobility quadrupole time-of-flight mass spectrometry; HILIC-UHPLC-Q-TOF MS, Hydrophilic Interaction Liquid Chromatography-Ultra-high-performance liquid chromatography quadrupole time-of-flight mass spectrometry; SOPC, 1-stearoyl-2-oleoyl-sn-glycerol 3-phosphocholine; ILD, interstitial lung disease; HPLC-MS, High-performance liquid chromatography-mass spectrometry; PAH, pulmonary arterial hypertension; H NMRS, proton nuclear magnetic resonance spectrometry; VLDL, very-low-density lipoprotein; LDL, low-density lopoprotein; PVDOMI, pulmonary vascular disease phenomics; JHSC, JohnsHopkins Scleroderma Center; IPAH, idiopathic pulmonary arterial hypertension; JP, joint pain; TE, telangiectasia.

In recent years, researchers have shifted their focus towards exploring the metabolic profile of SSc patients with pulmonary complications, offering new insights into the diagnosis and management of the disease. Within ILD patients, notable alterations in amino acid metabolism are observed. Contrasting with non-ILD patients, those with ILD exhibit elevated levels of specific amino acids (including Hcy, arginine, and valine) and fructosamine derived from branched-chain amino acids, while glycerophosphoethanolamine (e.g., phosphatidylethanolamine 36:3, 38:5, 38:6) and steroids (such as androstenolone 3-sulphate) are reduced (41, 44, 46, 48, 56). Moreover, a study distinguishing stable from progressive SSc-ILD patients revealed that metabolite levels like L-leucine, L-isoleucine, xanthine, and adenosine monophosphate could potentially serve as biomarkers to differentiate between the two phenotypes (43), offering additional insights into SSc-ILD biomarkers. Sun et al. further supported that glutamine metabolism stands out as a major metabolic pathway in SSc-ILD patients (44). Notably, proinflammatory metabolites like TMAO were significantly elevated in SSc-ILD patients with esophageal motility dysfunction, with a notable correlation between TMAO concentrations, N-terminal precursor brain natriuretic peptide (a cardiac involvement marker), and ILD severity (60). Amino acid levels such as valine, kynurenine, L-proline, and proline-histidine were also found to be higher in urine samples from ILD patients compared to those without ILD (48).

In patients with SSc-PAH, metabolic abnormalities likely play a pivotal role in the pathophysiology of PAH. Compared to SSc patients without PAH, those with PAH demonstrate increased levels of specific amino acids and their derivatives (e.g., alanine), lipids (e.g., very-low-density lipoprotein, low-density lipoprotein), and lactic acid. Conversely, there is a reduction in levels of other amino acids and their derivatives (e.g., γ-amino-butyric acid, arginine, phenylalanine, tyrosine, histidine, glycine, glutamate, glutamine, betaine), as well as choline, creatine, and glucose (62). When compared with patients with Idiopathic Pulmonary Arterial Hypertension (IPAH), individuals with SSc-PAH exhibit significantly higher levels of fatty acid metabolism (e.g., lignoceric acid, nervonic acid), steroid hormone metabolism (11-testosterone and 17β estradiol), and arachidonic acid metabolism (novel classes of eicosanoids, prostaglandins F2α, and leukotriene B4), indicating distinct metabolic profiles between the two conditions (63). Various studies have consistently demonstrated lowered Trp levels in SSc patients (39, 41–43). Additionally, a study by Simpson et al. revealed significantly elevated levels of Kyn and Kyn/Trp ratio in wedge veins compared to mixed vein samples from SSc-PAH patients (64), hinting at the potential involvement of the Kyn pathway metabolism in PAH development. Moreover, elevated levels of L-NAME and decreased levels of L-arginine in SSc-PAH patients compared to those with SSc alone suggest impaired vasodilation functions (65), potentially contributing to PAH development. Given that L-arginine serves as a precursor to NO, its reduction might exacerbate NO deficiency, impacting vascular tone and blood flow. These metabolites seem closely linked to the pathophysiological mechanisms of SSc-PAH. Notably, significant differences in Hcy concentrations have been observed in SSc patients with macrovascular issues and thromboembolic events (46, 47). Elevated plasma Hcy levels (36, 37) in SSc patients are associated with complications like vascular embolic events, PAH, finger ulcers, limb osteolysis, and ILD (46, 47). Elevated Hcy levels in the context of SSc could potentially serve as a serum marker for the disease.

There is limited data on metabolite studies related to autoantibody positivity in SSc. In a plasma sample analysis, elevated levels of Kyn and decreased levels of Trp were observed in patients positive for ARA compared to those with ACA and anti-Scl70 antibodies (39). This finding suggests a potential influence of Kyn and its metabolites on B lymphocyte differentiation and activation, contributing to the immunomodulatory processes in SSc. A comprehensive review of the Kyn and Kyn/Trp levels in different SSc subtypes revealed heightened concentrations in SSc-associated PAH, dcSSc, and ARA subtypes (39, 42, 64), indicating that these metabolites from the Kyn/Trp pathway could serve as biomarkers for early disease detection and stratification, particularly in vascular-associated SSc lesions. Moreover, Russo et al. highlighted that patients positive for anti-Scl-70 antibodies presented increased levels of propionic acid in fecal samples, while ACA-positive patients exhibited elevated hexanoic acid levels in their fecal samples. Plasma valeric acid levels tended to be higher in ACA-positive patients (24). In other subtypes of SSc, a plasma sample analysis revealed elevated levels of glutamate, sarcosine, proline, tyrosine, 4-methylhistidine, and ornithine in patients with SSc-associated calcinosis. Conversely, patients experiencing SSc joint pain had increased ornithine and 1-methylhistidine levels but decreased glutamine levels (41). Collectively, these findings suggest that the observed metabolite abnormalities in SSc patients may be linked to autoimmune dysregulations in SSc.

## 4 Association of GM and metabolites in SSc and potential molecular mechanisms

Exploring the involvement of GM and their metabolites in the development of skin and lung complications in SSc has recently advanced through proposed mechanisms like the gut-skin axis (66, 67) and the gut-lung axis (68, 69). Despite these advances, the precise mechanisms of action remain ambiguous. In this context, we address the specific or potential metabolic interactions between gut flora and SSc, drawing from existing literature evidence.

### 4.1 Association of GM with Trp metabolism

Within the extensive investigations on amino acid metabolism, notable changes in Trp metabolism emerge, characterized by reduced Trp levels in SSc patients (39, 41–43). Trp, an indispensable aromatic amino acid, holds significance as a biosynthetic precursor for a myriad of microbial and host metabolites despite being the least abundant amino acid in proteins and cells (70). In the GIT, Trp metabolism encompasses three primary pathways (71) (1): direct conversion by gut microbes into molecules like aryl hydrocarbon receptor (AhR) ligands (2); catalytic conversion to Kyn by indoleamine 2,3-dioxygenase (IDO) 1 in immune and epithelial cells (3); transformation into 5-hydroxytryptamine by Trp hydroxylase 1 in enterochromaffin cells. This metabolic cascade yields various bioactive compounds that modulate key physiological functions spanning inflammation, metabolism, immune response, and neurological activity (72). Notably, elevated levels of Kyn and Kyn/Trp are observed in specific SSc subtypes, including SSc-PAH, dcSSc, and ARA-positive isoforms (39, 42, 64). The involvement of the Kyn pathway and its metabolites in inhibiting T-cell proliferation and inducing T-cell apoptosis has been underscored (73, 74). Most microbiota, aside from viruses and archaea, metabolize Trp through diverse pathways to produce bioactive compounds (75, 76). Montgomery et al. identified that the gut commensal Lactobacillus reuteri metabolizes Trp to generate various derivatives, activating AhR and potentially enhancing the T-cell response to produce IL-17, thereby regulating autoimmunity (77). Additionally, commensal bacteria may combat viral infections by AhR activation (78). A synthesis of existing literature highlights that several commensal bacteria exhibit anomalies in SSc patients, with Lactobacillus showing elevated relative abundance (10, 19, 21, 22, 25, 28, 29), suggesting that intestinal commensals could influence the immune system in SSc by modulating host Trp production and its metabolites, thereby eliciting diverse responses in epithelial and immune cell populations.

### 4.2 Association of GM with lipid metabolism

While previous studies on the GM-metabolome co-axis have predominantly explored water-soluble, polar metabolites like Trp catabolic metabolites and amino acids, the microbial-host-lipid co-axis has not received adequate attention. In SSc, the relationship between GM and lipid metabolism is variably interconnected. Ottria et al. conducted experiments applying etoposide, a carnitine transporter protein inhibitor, to incubated dendritic cells (DCs) from healthy individuals and those with SSc, revealing inhibition of proinflammatory cytokine production through the suppression of fatty acid oxidation. This suggests a potential role of fatty acids in augmenting inflammation in SSc patients (54). Current emerging evidence highlights the involvement of lipids in autoimmune diseases and IBD (79). Recent research indicates the significance of intestinal commensal bacteria, such as Bacteroides, in sphingolipid production for maintaining intestinal homeostasis and symbiosis (80). Furthermore, the Lactobacillus genus appears to be closely associated with fatty acyls (81–83). Conversely, commensal genera believed to possess anti-inflammatory properties, such as Bacteroides, demonstrate reduced relative abundance in SSc (21, 23, 31). Additionally, commensal genera recognized for their anti-inflammatory effects, like Anabaena spp., also exhibit diminished relative abundance in SSc.

Butyrate, a short-chain fatty acid (SCFA), serves as a crucial energy source for intestinal epithelial cells, bolsters various components of the colonic defense barrier, reduces oxidative stress, and exerts anti-inflammatory and immunomodulatory effects (84). Studies have consistently highlighted the significant role of SCFAs in lung disease by inhibiting the histone deacetylase enzyme and maintaining intrapulmonary homeostasis (85). Butyrate, propionate, and acetate collectively regulate intrapulmonary homeostasis and immunity (86, 87). Faecalibacterium and Clostridium are prevalent in the GIT of healthy individuals, fortifying the epithelial barrier through butyrate production and aiding in mucosal inflammation regulation. Yet, the occurrence of these beneficial commensal genera is reduced in SSc. Several investigations emphasize the decline of butyrate-producing commensal bacteria in both SSc and IgG4-RD contexts (20, 31). Studies confirm that Desulfovibrio, a pathogenic bacterium linked to intestinal dysregulation in SSc, induces inflammatory responses in the ileum and colon (88, 89). Desulfovibrio interacts with amino acid metabolism and glycerophospholipids, forming heterodimers with SCFAs such as butyrate and ultimately contributing to intestinal dysregulation and inflammation (20). Pathogenic bacteria-mediated host pathophysiology is driven not only by the production of harmful metabolites but also by a reduction in beneficial metabolites. Patients with SSc exhibit elevated levels of pro-inflammatory bacteria like Desulfovibrio and reduced levels of protective butyrate, indicating a disturbance in the balance of GM (20, 31). Consequently, the GM in SSc may impact the host’s immune response by influencing SCFA metabolism, particularly the butyrate pathway, thereby exacerbating inflammatory and fibrotic processes.

### 4.3 The potential molecular mechanisms of GM and metabolites in SSc

SSc is characterized by three primary pathogenic mechanisms: microvascular damage, immune dysregulation, and multiorgan fibrosis. T-cell subsets play a crucial role in maintaining protective immunity against pathogens while regulating inflammatory responses to self and microbial antigens. Guided by environmental cues and antigen-presenting cells, CD4^+^ T helper (Th) cells differentiate into distinct subsets like Th1, Th2, Th17, and regulatory T cells (90). The equilibrium of these cell populations, particularly the Th17/Treg balance, is pivotal in the transition from homeostasis to disease and is significantly shaped by the GM (91, 92). Disruption of the GM can disturb the intricate balance between microbiota and the immune system, leading to inflammation and fibrosis and potentially contributing to the development and progression of SSc. Fibrosis, a complex pathological process, involves the abnormal accumulation of extracellular matrix (ECM) components post-tissue damage (93). An imbalance in the GM can result in the accumulation of harmful compounds and depletion of beneficial substances such as SCFAs (94, 95). This dysbiosis is often associated with compromised intestinal barrier integrity, facilitating the translocation of bacteria and their byproducts into the bloodstream, triggering systemic immune and inflammatory responses that may lead to tissue damage, either directly or indirectly (96).

**Figure 1:**
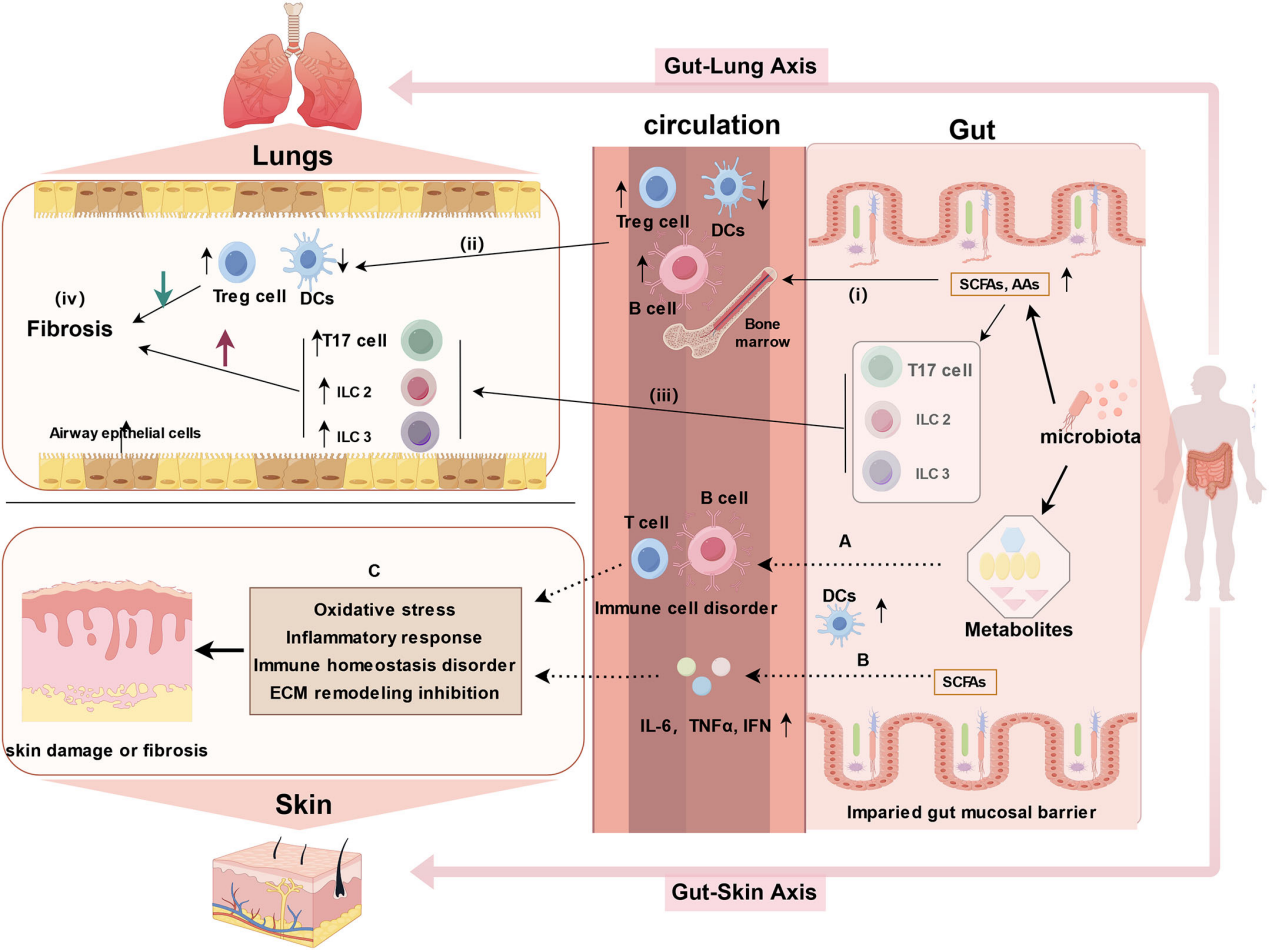
Potential mechanisms of the gut-lung axis and gut-skin axis in SSc (created by figdraw). 1. gut-lung axis: (i) Microbial metabolites, such as SCFAs and amino acids (AAs), enter the bloodstream and influence the development of immune cells; (ii) Activation of bone marrow-derived immune cells triggers immune responses in the lungs; (iii) Cells migrating from the gut, including innate lymphoid cells 2/3 (ILC2/3) and Th17 cells, can affect pulmonary immunity by migrating to the lungs; and (iv) hence, microorganisms and metabolites influence the course of pulmonary fibrosis through the intermediate link of immune disorders. 2. gut-skin axis: **(A)** Gut microbes and metabolites alter immune cells (e.g., impairing T cell differentiation and promoting B cell hyperresponsiveness), which in turn affect skin disorders through circulation; **(B)** SCFAs upregulate DCs and enhance the production of IL-6, IFN, and tumor necrosis factor (TNF); **(C)** The combined effects of A and B leads to skin lesions in SSc through the induction of oxidative stress, inflammatory response, disruption of immune homeostasis, and inhibition of ECM remodeling. DCs: dendritic cells.

Chronic inflammation is recognized as the primary trigger of intestinal fibrosis. Prolonged exposure to chronic inflammatory stimuli induces the activation and proliferation of mesenchymal cells (e.g., fibroblasts, myofibroblasts, or smooth muscle cells) to produce ECM, ultimately leading to fibrosis. PF is a common complication and a poor prognostic indicator in advanced stages of SSc-ILD. PF is primarily marked by lung inflammation and excessive ECM deposition, resulting in structural alterations in lung tissue, such as thickening and scarring of the lung parenchyma, particularly concentrated in the interstitium (97). Recent research has shed light on the existence of a bidirectional gut-lung axis (68, 69), where microorganisms (98–100), immune functions (98, 101, 102), and metabolites (69, 102) are exchanged through the blood and lymphatic system, influencing the pathophysiological processes of the disease. Although in-depth studies on the gut-lung axis in SSc are limited, evidence suggests that various intestinal microbial metabolites, such as amino acids, SCFAs, and bile acids, can impact fibroblasts, myofibroblasts, extracellular matrix accumulation, immune regulation, and other pathways, potentially contributing to ILD or PF (103). Amino acids are prevalent intestinal metabolites in SSc patients, including compounds like arginine (40, 62) and glutamine (38, 41, 44). Arginine, in particular, has been implicated in collagen deposition, apoptosis, and ammonia elimination in individuals with idiopathic PF (IPF). Cellular experiments have shown that glutamine contributes to apoptosis resistance in lung fibroblasts derived from IPF patients. Reduced glutamine metabolism increases the susceptibility of IPF fibroblasts to Fas ligand (FasL)-induced apoptosis, downregulates the expression of anti-apoptotic genes, and induces epigenetic changes in these cells (104). The gut-lung axis involves multiple pathways of immune communication. For example, SCFAs and amino acids circulate through the bloodstreams activating bone marrow-derived immune cells, which subsequently influence immune cell development and trigger immune responses in the lungs. Additionally, innate lymphocytes 2/3 (ILC 2/3) and Th 17 cells may migrate from the gut to the lung, impacting pulmonary immune responses (**Figure 1**) (105–107). This emerging perspective on the gut-lung axis offers valuable insights into the cellular mechanisms underlying PF and provides a novel framework for studying the pathogenesis of SSc-ILD.

**Figure 2:**
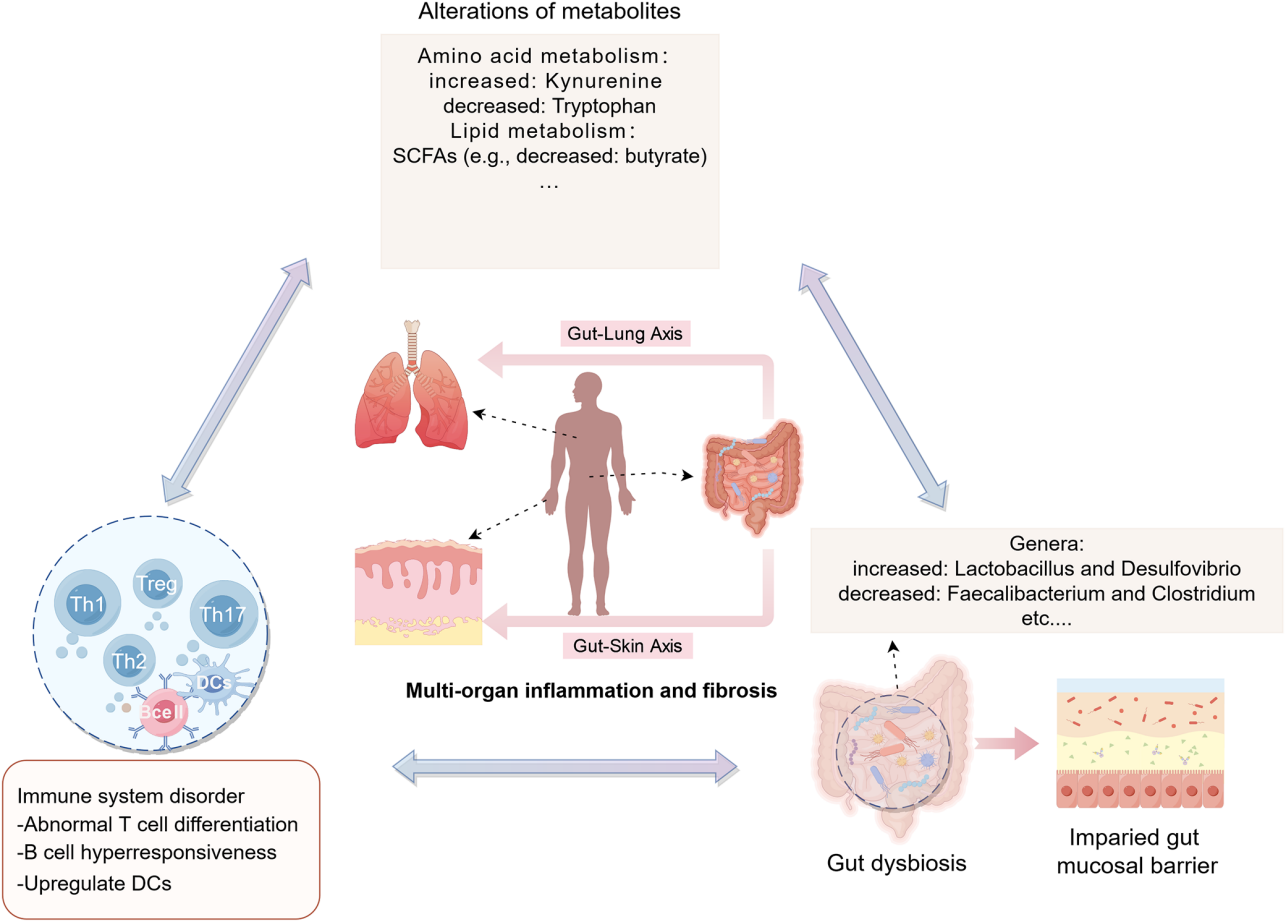
Potential mechanisms of the gut microbiota and its metabolism in SSc (created by figdraw). In SSc, gut dysbiosis can compromise the integrity of the intestinal barrier, leading to increased intestinal permeability. Consequently, this breach permits the entry of abnormal metabolites (e.g., kynurenine, etc.) and other contents from the gut lumen into the bloodstream. This influx, facilitated by the intestinal-skin/lung axis pathway, has the potential to exacerbate autoimmune disorders and inflammation, culminating in damage to target organs like the skin, intestines, and lungs. DCs: dendritic cells.

Furthermore, a similar bidirectional relationship is observed between the gut and skin, referred to as the gut-skin axis. Skin and gut both are active, complex immunological and neuroendocrine organs, impairment of the intestinal barrier permits the passage of intestinal microbiota and metabolites into the bloodstream, affecting autoimmune responses and inflammatory conditions in skin (17, 67). Communication between the gut and skin occurs through immune cross-talk. Dysbiotic gut microbes, toxic products, neurotransmitters, and altered immune cells, including impaired T cell differentiation and hyperresponsive B cells, circulate through the bloodstream, contributing to dysbiotic skin conditions (**Figure 1A**) (108). SCFAs, as metabolic by-products, can upregulate DCs and enhance the production of proinflammatory cytokines, which in turn trigger an inflammatory response (**Figure 1B**). Probiotics, including *Nitrobacter*, *Lactobacillus*, and *Bifidobacterium*, help restore intestinal homeostasis by correcting imbalances in the gut microbiota and repairing damage to the intestinal mucosal barrier (109). These probiotics can improve skin diseases by inhibiting oxidative stress, reducing inflammatory responses, and restoring immune homeostasis, as well as modulating ECM remodeling (108, 110). Thus, the regulation of skin conditions via the gut-skin axis may involve mechanisms such as the inhibition of oxidative stress, suppression of inflammatory responses, restoration of immune homeostasis, and inhibition of ECM remodeling (**Figure 1C**). Numerous studies have demonstrated that disruptions in the gut microbiota can contribute to the development of various skin diseases, such as atopic dermatitis (111) and psoriasis (112, 113). The gut-skin microbiome axis may also play a role in systemic autoimmune diseases, with studies suggesting that skin commensals are implicated in systemic lupus erythematosus (114). Recent findings by E. Russo et al. suggest that specific circulating autoantibodies may guide the differential dynamics of the gut-skin microbiota axis in SSc subsets. Notably, ACA-positive and anti-Scl70-positive patients exhibit distinct microbial signatures in both affected skin and gut regions, along with differing profiles of serum and fecal free fatty acids (24). This accumulating evidence underscores the significant impact of gut dysbiosis and metabolic disruptions in the progression of fibrosis in the skin, gut, and lungs in SSc (**Figure 2**).

## 5 Conclusion and prospects

In conclusion, GM and metabolism display diverse abnormalities in SSc and are linked to symptoms and disease subtypes. Nevertheless, there is a paucity of definitive studies regarding the significance of pathogenic or probiotic bacteria and their metabolites in SSc. The interplay among microbiota, metabolites, and the immune system may act as a potential triggering factor, influencing the overall health of the host through pathways such as the gut-skin and gut-lung axis. Despite encountering challenges, the integration of multi-omics for SSc analysis holds significant promise. The successful merging of macro-genomics and metabolomics has revealed insights into the relationship between gene regulation, microbes, and metabolism within the microbiome, although additional validation studies are required. Integrated multi-omics data analysis provides a more comprehensive understanding of DNA identification and metabolite functions in the microbiome, thus enhancing the informative value of microbial research in SSc.
